# BLIGHTSIM: A New Potato Late Blight Model Simulating the Response of *Phytophthora infestans* to Diurnal Temperature and Humidity Fluctuations in Relation to Climate Change

**DOI:** 10.3390/pathogens9080659

**Published:** 2020-08-15

**Authors:** Hossein A. Narouei-Khandan, Shankar K. Shakya, Karen A. Garrett, Erica M. Goss, Nicholas S. Dufault, Jorge L. Andrade-Piedra, Senthold Asseng, Daniel Wallach, Ariena H.C van Bruggen

**Affiliations:** 1 Department of Plant Pathology, University of Florida, 1450 Fifield Hall, P.O. Box 110680, Gainesville, FL 32611-0680, USA; sshakya@ballhort.com (S.K.S.); karengarrett@ufl.edu (K.A.G.); emgoss@ufl.edu (E.M.G.); nsdufault@ufl.edu (N.S.D.); ahcvanbruggen@ufl.edu (A.H.C.v.B.); 2 Emerging Pathogens Institute, University of Florida, Gainesville, 2055 Mowry Road, P.O. Box 100009, Gainesville, FL 32610, USA; 3 Ministry for Primary Industries, P.O. Box 2526, Wellington 6146, New Zealand; 4 Food Systems Institute, University of Florida, P.O. Box 110180, Gainesville, FL 32611-0180, USA; 5 International Potato Center (CIP) and CGIAR Research Program on Roots Tubers and Bananas (RTB), P.O. Box 1558, Lima 12, Peru; j.andrade@cgiar.org; 6 Department of Agricultural and Biological Engineering, University of Florida, 224 Frazier Rogers Hall, P.O. Box 110570, Gainesville, FL 32611-0570, USA; sasseng@ufl.edu; 7 Institut National de la Recherche Agronomique (INRA), UMR AGIR, BP 52627, 31326 Castanet Tolosan Cedex, France; Daniel.wallach@inrae.fr

**Keywords:** Simulation model, SEIR model, R, relative reproductive rate, lesion growth, oscillating temperatures

## Abstract

Temperature response curves under diurnal oscillating temperatures differ from those under constant conditions for all stages of the *Phytophthora infestans* infection cycle on potatoes. We developed a mechanistic model (BLIGHTSIM) with an hourly time step to simulate late blight under fluctuating environmental conditions and predict late blight epidemics in potato fields. BLIGHTSIM is a modified susceptible (S), latent (L), infectious (I) and removed (R) compartmental model with hourly temperature and relative humidity as driving variables. The model was calibrated with growth chamber data covering one infection cycle and validated with field data from Ecuador. The model provided a good fit to all data sets evaluated. There was a significant interaction between average temperature and amplitude in their effects on the area under the disease progress curve (AUDPC) as predicted from growth chamber data on a single infection cycle. BLIGHTSIM can be incorporated in a potato growth model to study effects of diurnal temperature range on late blight impact under climate change scenarios.

## 1. Introduction

Potato late blight, caused by *Phytophthora infestans*, may be the most important plant disease worldwide since it was associated with the infamous Irish Potato Famine in the 1840s. Climate change is expected to lead to other plant disease outbreaks [1] that may result in food shortages and mass migrations similar to the Irish Potato Famine. Late blight has been the subject of many empirical forecasting models [2,3,4,5,6,7,8,9] as well as mechanistic simulation models [3,10,11,12,13,14,15,16,17,18,19,20,21,22], but few are focused on the potential effects of climate change [9,23]. The empirical forecasting systems are often derived from BLITECAST [24], and are based on the accumulation of late blight risk units under daily temperature and humidity conditions summarized from hourly data [5,6,7,9,14,23]. The forecasts were improved when artificial neural network models with hourly weather data were used for the predictions [2]. 

Most simulation models consist of successive stages in the infection cycle [10,12,16,17,20,21,22,25] or spore release, survival and aerial transport [11,13,15,19]. The first and most widely used simulation model is LATEBLIGHT [12]. This is a matrix model where lesions pass through 15 age classes and at the same time expand in area. The age classes represent different stages of pathogen development from sporangial or zoospore germination to microscopic lesion, expanded lesion, sporulating lesion and necrotic lesion after spore dispersal. Daily composite measures of temperature, relative humidity (RH) and leaf wetness are the driving variables for sporulation and infection, but not for the latent period. LATEBLIGHT was modified by several authors, for example to account for effects of fungicides and potato cultivars [18]. In an approach similar to that of Berger and Jones [26]. Andrade-Piedra et al. [27] modified the temperature effects on lesion expansion and sporulation, and added temperature effects on the latent period. The modified model was programmed using the Statistical Analysis System (SAS Institute Inc., Cary, NC, USA) and was referred to as LB2004. This model was evaluated for a wide range of ecoclimates with the ultimate goal to predict global late blight risk under climate change conditions [27]. Very few other models have been used to predict potential effects of climate change on late blight [9,23], and these were empirical models based on BLITECAST or SimCast, a modification of BLITECAST using average temperatures during the hours per day with high RH [3,5].

Global climate change, as predicted by general circulation models, will likely have profound effects on plant disease development [1,28,29,30,31,32]. Current climate predictions include not only changes in average temperature and humidity but also in their daily amplitudes [33]. Trends in diurnal temperature range (DTR) could possibly be a key feature of climate change [34]. Recent climate change models predicted that DTR can range from 5˚C (near-coastal regions at high latitudes) up to 21˚C (in dry and/or high altitude regions) [35]. On average, diurnal amplitudes in temperature are expected to decrease [35], although a recent increase in diurnal variation has been documented [36]. At high altitudes, for example in the high Andes, the DTR is expected to increase because reduced cloud cover may raise the daily maximum temperature more than the daily minimum temperature [37]. Alternatively, under climate change, the expected minimum temperature would be reduced less than expected by a reduction in cloud cover. Due to the great variations in climate and complex interrelationships between climate, plant pathogens, their hosts and field managers, prediction of plant disease development under climate change is challenging [28]. 

Nevertheless, several attempts have been made to predict how climate change might affect particular plant pathogens or pathogen ecotypes [23,30,32]. These predictions have been made based on average changes in temperature and humidity in large climatic and ecological regions [23]. When aggregated monthly weather data were used as inputs in an empirical forecasting model, the global mean effect of climate change on potato late blight risk was predicted to be small, with some hot spots for increased disease risk and some locations with decreased risk [23]. However, plant disease development can be strongly affected by diurnal variation in temperature, RH, leaf wetness and light conditions [28,38,39,40,41,42]. This is certainly true for potato late blight development, which differs significantly under diurnal oscillating versus constant temperatures with the same means [40]. Shakya et al. [40] showed that at relatively low average temperatures (<17 °C), the incubation and latency progression rates of *P. infestans* were faster under oscillating than under constant conditions, while these rates were slower under oscillating conditions at average temperatures >17 °C. Moreover, small oscillations in temperature (+5 °C) increased infection efficiency, lesion growth rate and sporulation, whereas large oscillations (+10 °C) reduced these variables below the constant temperature values [40]. Such differences in the response of epidemic components to oscillating versus constant temperatures will likely have profound effects on predictions made for plant disease development under global climate change [28,43].

For prediction of potential effects of global climate change on potato late blight and yield, a mechanistic late blight model would need to be coupled to a physiological potato growth model [16,30,44]. Such a late blight model would need to be relatively simple, so that it could easily be incorporated in a potato growth model. The LATEBLIGHT and LB2004 models are quite complex due to the many lesion categories and separate categories for sporangia and zoospore production and germination [12,27]. SEIR models, with potential infection sites that are successively susceptible, exposed, infectious and removed (SEIR) [45], can be quite simple and easily integrated into crop growth models. Only a few SEIR models have been published for late blight development [10,22]. All late blight simulation models have daily time steps and use average weather, while several researchers have indicated that an hourly time step would be preferable, as the various epidemic components respond strongly to diurnal temperature variations [16,40,46]. A model with an hourly time step would also enable testing the hypothesis that, besides average temperature, the diurnal amplitude would affect epidemic development and the area under the disease progress curve (AUDPC). 

The main objective of this research was to develop and validate a simple modified SEIR model that could simulate both new infections and lesion growth with an hourly time step, so that effects of diurnal temperature and humidity oscillations on disease development could be simulated accurately. The second objective was to test the hypothesis that both diurnal amplitudes and average temperatures determine the AUDPC. A third objective was to investigate whether the initiation time of day would affect the outcome of a model with an hourly time step. Model calibration was based on lesion development data obtained from growth chamber experiments [40]. Further calibration and validation of the model was accomplished with field data from Ecuador [47]. Hypothesis testing was done by using modeled temperature and humidity oscillations based on modified sine waves as input variables, combined with parameter estimates from field data. The simulation model was written in the removed (R) programming environment and named BLIGHTSIM.

## 2. Results

### 2.1. Model Development

**Figure 1:**
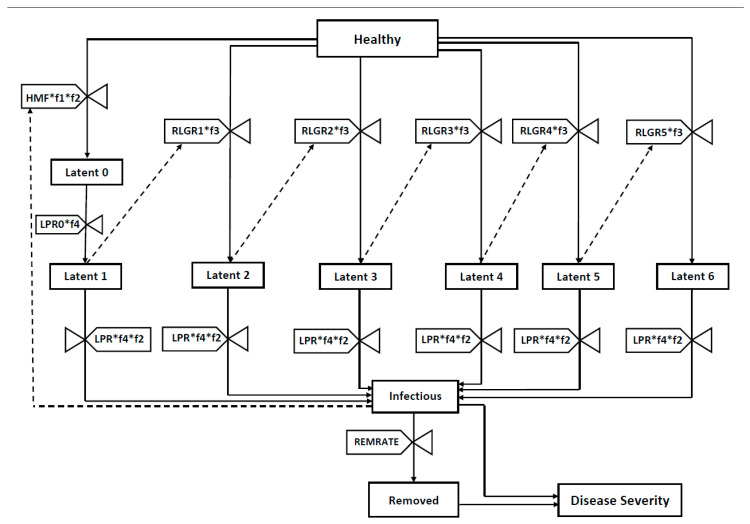
Relational diagram of the BLIGHTSIM model for simulation of potato late blight (*Phytophthora infestans*) with all compartments and rate variables. Healthy tissue units may transition to latent tissue units through parallel pathways: through infection by spores (transitioning from Healthy to Latent 0) or through “lesion growth”, where a healthy tissue unit is located adjacent to rings of expanding latent units, Latent 1, Latent 2 and Latent 3 (transitioning from Healthy to Latent 2, Latent 3 or Latent 4, respectively). For a description of abbreviations see Table 1.

We designed a simple SEIR compartmental model, called BLIGHTSIM which simulates seasonal epidemic development in a few seconds on a typical personal computer (Figure 1). The compartments are healthy sites (H), latent sites (Lx), infectious sites (I) and removed sites (R). Latently infected sites are divided into latent compartments: L0 is formed from infection of H by spores and turns into L1 towards the end of the latent period; the other latent compartments (L2, L3, L4, L5 and L6) originate from “lesion growth” on five successive days (See the Methods section for more details).

### 2.2. Temperature and Relative Humidity Response Curves

**Figure 2:**
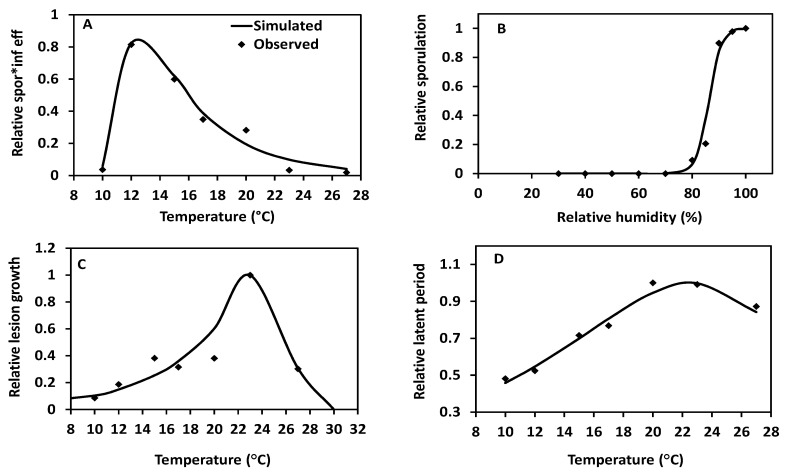
Response curves for parameter estimates used in the model BLIGHTSIM. All data except those in B were obtained from growth chamber experiments performed on potato cv Red LaSoda using an isolate of *Phytophthora infestans* clone US-23 [40]. The data for A, C and D were fitted with a four-parameter thermodynamic model (Table S1). The data for B were fitted using a logistic regression model is SAS (version 9.4). **A**, effect of temperature on relative sporulation*infection efficiency; **B**, effect of relative humidity on sporulation *of P. infestans* race 4,10,11 on potato cv Bintje [48]; **C**, effect of temperature on relative radial lesion growth rate; and **D**, effect of temperature on relative latency progression rate.

Based on data from growth chamber experiments [40], a right-skewed response curve was obtained for relative sporulation*infection efficiency (Figure 2A), similar to the individual response curves for sporulation and infection efficiency (see Figure 1B and Figure 3D in [40]). The relative sporulation*infection efficiency was maximal at 12 °C and gradually reduced beyond that temperature (Figure 2A). The sporulation probability at 15 °C plotted versus RH resulted in a saturation curve reaching the maximum above 95% RH (Figure 2B). A left-skewed response curve was obtained for relative lesion growth rate versus temperature with an optimum at 24 °C (Figure 2C). Fitting the relative latency progression rate to the thermodynamic model also resulted in a left-response curve with an optimum at 22–23 °C (Figure 2D). The parameter values for the thermodynamic and logistic models are given in Table S1.

### 2.3. Model Calibration with Growth Chamber Data

The initial proportion of latent sites (L0) was adjusted for each average temperature and amplitude combination to simulate the disease severity over time (Table 1). Under constant temperature conditions, the estimated L0 was lowest at the lowest temperature evaluated, an average temperature of 10 °C, maximal at 12 to 15 °C and lower at higher temperatures, reflecting the observed response curves for infection efficiency. When the temperature amplitude was 5 °C, the estimated L0 was relatively high at an average temperature of 10 °C, maximal at average 12°C, and very low at average temperatures equal to or above 20 °C, again reflecting the observed infection efficiency. When the temperature amplitude was 10 °C, the trend in estimated L0 values was similar to that under constant temperatures, but with a maximum at 20 to 23 °C.

### 2.4. Model Output Simulating Growth Chamber Conditions

**Figure 3:**
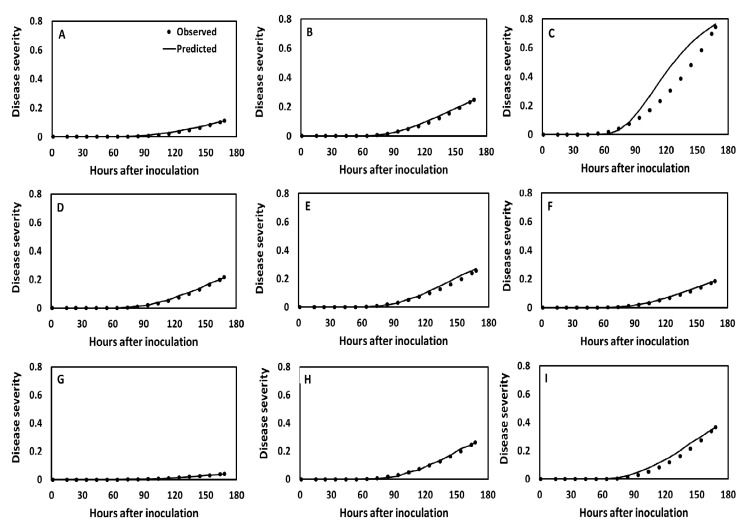
Observed (dots) and predicted (continuous line) disease progress curves of an isolate of *Phytophthora infestans* clone US-23 at different constant (A-C) and oscillating (D-I) temperatures (T). **A**, T = 12 °C; **B**, T = 17 °C; **C**, T = 23 °C; **D**, T = 12 ± 5 °C; **E**, T = 17 ± 5 °C; **F**, T = 23 ± 5 °C; **G**, T = 12 ± 10 °C; **H**, T = 17 ± 10 °C; and **I**, T = 23 ± 10 °C. The simulated disease progress curves were obtained with BLIGHTSIM and the observed disease severities in a growth chamber experiment at Gainesville, Florida.

When the model outputs were evaluated over a 168 h time course at seven constant temperatures ranging from 10 °C to 27 °C, and a good fit was obtained to the observed disease severity data (Figure 3A–C and Figure S1). The R^2^ of the simulated versus observed data was above 0.934 in all cases. The simulated disease severity increased at increasing average temperatures from 10 °C to 23 °C as observed in the growth chamber experiment and decreased beyond that temperature. The final observed and predicted disease severities were highest (74%) at 23 °C and lowest (1.7%) at 10 °C.

When simulations were evaluated for a 168h time course under oscillating temperatures with amplitude ±5 °C, excellent fits were obtained (Figure 3D–F and Figure S2), with R^2^ values above 0.97 for the simulated versus observed regression lines (Table 1). The simulated and observed final disease severities were lower under oscillating than under constant temperatures. The highest final disease severity under oscillating temperatures was 25% at 17 ± 5 °C and the lowest was 6% at 10 ± 5 °C.

When simulations were evaluated for a 168 h time course at oscillating temperatures ranging from 10 °C to 27 °C with amplitude ±10 °C (Figure 3G–I and Figure S3), the final disease severity was again low (up to 36%) compared to simulated disease severities under constant temperatures but higher than those at oscillating temperatures with amplitude ±5 °C. Disease severity increased with increasing average temperatures with amplitude 10 °C up to 23 °C and declined at higher average temperatures. Maximum disease severity was 36% at 23 ± 10 °C whereas minimum disease severity was 4% at 10 ± 10 °C.

### 2.5. Model Output Simulating Field Conditions

The estimated relative reproductive rate (RRR) values were slightly different for the two cultivars, i.e., 0.049 for Gabriela and 0.052 for Bolona. The proportions of latent sites calibrated to start the epidemics were very different for the two locations (Table 2). For La Tola, the proportion of initial inoculum was set at 0.000001 in 1997 and 1998, while for Cutuglahua it was estimated as 0.0007 in 1997 and 0.0001 in 1998 (Table 2). 

**Figure 4:**
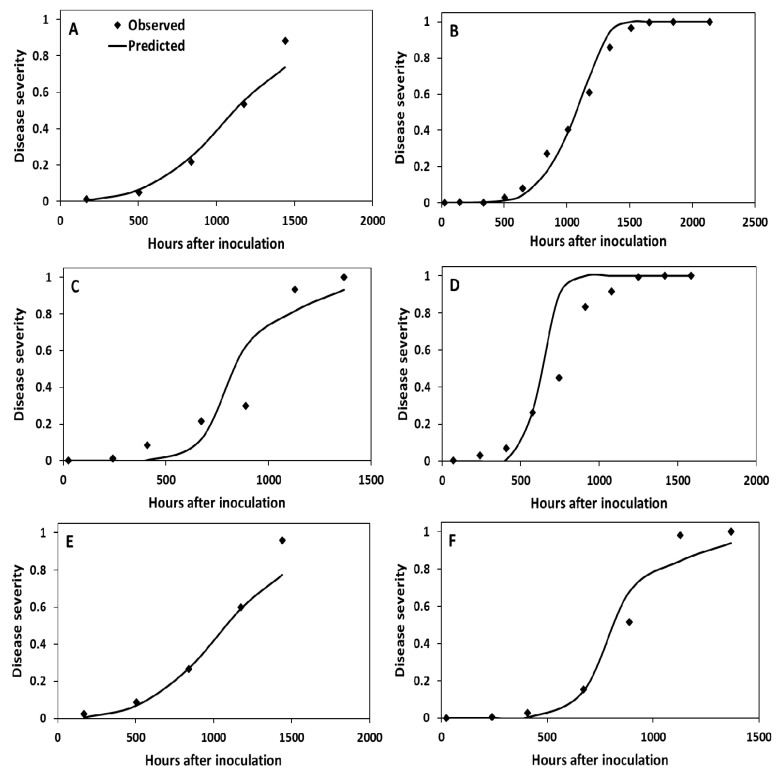
Observed (dots) and predicted (continuous line) disease progress curves for six late blight epidemics under field conditions at two locations, La Tola and Cutuglahua in Ecuador. **A**, model calibration for cultivar Gabriela at Cutuglahua in 1997; **B**, model validation for cultivar Gabriela at Cutuglahua in 1998; **C**, model calibration for cultivar Gabriela at La Tola in1997; **D**, model validation for cultivar Gabriela at La Tola in 1998; **E**, model calibration for cultivar Bolona at Cutuglahua in 1997; and **F**, model validation for cultivar Bolona at La Tola in 1997. The relative reproductive rates and initial proportion of latent sites are given in Table 2.

When the model was run for Cutuglahua in 1997 (1465 h) a very good fit (R^2^ = 0.976) was achieved to the observed disease severities for cultivar Gabriela (Figure 4A). When the same parameter values were used together with the weather data of 1998, the fit was also very good (R^2^ = 0.991) for the observed data for the same cultivar in Cutuglahua (Figure 4B), although the proportion of initial latent sites was lower in 1998 than in 1997 (0.0001 versus 0.0007) (Table 2). When the model was calibrated for cultivar Gabriela at La Tola in 1997, only a fairly good fit (R^2^ = 0.875) was observed (Figure 4C). A fairly good fit (R^2^ = 0.897) was also observed when the model was validated with 1998 data (Figure 4D). 

### 2.6. Scenario Testing 

**Figure 5:**
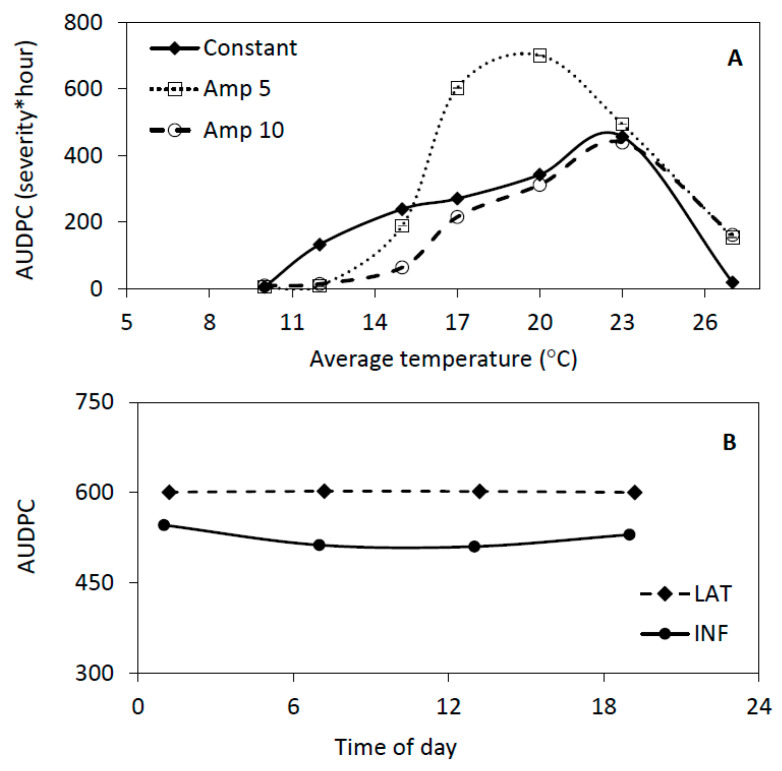
The effect of constant and oscillating temperatures on the average simulated area under the disease progress curve (AUDPC) values (3 replications) of late blight development over 1200 h.

For the first scenario set, there was an obvious interaction between average temperature and temperature amplitude (Figure 5). According to the repeated measures regression analysis the interaction was significant (*p* = 0.012). The amplitude effect was greater (*p* = 0.007) than the effect of average temperature (*p* = 0.034). The highest AUDPC was observed at average temperatures of 17 °C to 20 °C with an amplitude of ±5˚C (Figure 5). Under constant and widely oscillating (±10 °C) conditions, the maximum AUDPC was attained at 23 °C average temperatures. In general, the lowest AUDPC was obtained when the daily temperature amplitude was ±10 °C. 

The interaction between the simulation starting time of day and the initialization of the model runs with the proportion of latent or infectious sites was less obvious but significant (*p* < 0.0001). The AUDPC values were not significantly higher (*p* = 0.66) when the runs were initialized with latent sites rather than infectious sites, and there was only a starting time effect (*p* < 0.0001) when the model was initialized with infectious sites. In this last case, the AUDPCs were slightly lower when the model runs started during the day (7:00 am or 1:00 pm) than when they started in the evening (7:00 pm) or at night (1:00 am) (Figure S4).

## 3. Discussion

The primary objective of this research was to develop a relatively simple simulation model for potato late blight that uses hourly temperature and RH data as driving variables and provides hourly disease severity output. We succeeded at accurately simulating late blight disease progression at different average and amplitude combinations of daily temperatures using the new model BLIGHTSIM. This is a simple modified SEIR model with healthy, latent, infectious and removed sites and parallel pathways for expanding latent sites due to “lesion growth”, which is limited in time rather than space in BLIGHTSIM. The limitation of “lesion growth” in time has been used before [12], but in BLIGHTSIM this limitation is obtained in a different way compared to LATEBLIGHT [12]. In BLIGHTSIM, latent sites “grow radially” into healthy tissue during the last day before becoming infectious, comparable to lesion growth in the simulation model by Savary et al. [49]. Each lesion grows with a constant radial growth rate from its latent edge as in LB2004 [27]. Other authors have also assumed that lesions grow at a constant radial growth rate (mm.day^−1^) [20,22]. However, in BLIGHTSIM constant radial growth rates (mm.h^−1^) are converted to two-dimensional relative lesion growth rates (h^−1^) that decrease as lesions expand. 

To simulate the results obtained in growth chambers, where inoculation took place at a particular time, a delay function was needed. A simple delay in the latent period sufficed to obtain simulations reproducing the observed data. A delay function was not needed for the field data, because generations overlap, and the exact time of initial infection was not known. Taking the inverse of the latent and infectious periods as latency and infectiousness progression rates was a simple and adequate solution for simulating the delays under field conditions. This approach has often been taken for simulations of biological systems [45]. 

The model BLIGHTSIM provided an excellent fit to most of the observed disease severities in growth chamber experiments [40]. The simulated disease severities increased more slowly under oscillating (+5 °C) than constant temperatures at average temperatures above 17 °C. This could be explained by lower latency and incubation progression rates and initial number of lesions under oscillating than under constant temperatures above 17 °C [40]. When simulation results under constant versus oscillating temperatures were compared using model parameters estimated from field conditions, the simulated AUDPC values were significantly greater for oscillating conditions with an amplitude of 5 °C compared to constant temperatures at average temperatures above 17 °C. This difference can be attributed to a greater sporulation rate and lesion growth rate under oscillating conditions at relatively high average temperatures, outweighing reduced infection efficiencies and latency progression rates [40]. Despite the observed differences between oscillating and constant temperatures, the parameters of the constant temperature response curves were used successfully in BLIGHTSIM, resulting in simulated AUDPC values comparable to those obtained under oscillating conditions in the growth chamber (Figure 5; [40]). These results suggest that *P. infestans* responds instantaneously to the temperature at any moment, demonstrating the utility of models with an hourly time step. The simulation results also showed that the development of potato late blight is strongly affected by diurnal oscillations rather than daily averages as was observed previously for lettuce downy mildew [38,43]. 

In the field, spore production, dispersal and survival are strongly affected by various environmental conditions, including temperature and RH [41,42,50,51]. The transition from latency to infectiousness, i.e., initiation of sporulation, and RRR, which includes spore release and dilution as well as infection, depends on temperature and RH in BLIGHTSIM. RH is used as a proxy for leaf wetness, since leaf wetness data often are not available. This proxy has been used in many late blight forecasting models [5,6,7,9,14,23]. The approximation, based on the response of *P*. *infestans* to RH as determined by Harrison and Lowe [48], appeared to be adequate for this purpose, considering the very good fits of BLIGHTSIM to the field data. 

BLIGHTSIM was calibrated and validated with field data from Ecuador. The weather data were the same as those used in LB2004 by [47], except that in BLIGHTSIM hourly data were used instead of aggregated daily data. The simulation results frequently were better with BLIGHTSIM than with LB2004. For Cutuglahua 1997 (cultivar Gabriella), BLIGHTSIM showed a very good fit to calibration data, whereas LB2004 reached maximum disease severity 20 days before the observed maximum. BLIGHTSIM provided an excellent fit against validation data from the same location and cultivar in 1998, while LB2004 again predicted maximum severity 20 days before the observed maximum. For cultivar Gabriela at La Tola in 1997, both models performed reasonably well, although weather data for 384 h were estimated using regression analysis on weather data from a nearby weather station. Although BLIGHTSIM with its hourly time step is expected to be more sensitive to missing data compared to LB2004 with a daily time step, BLIGHTSIM performed better than LB2004, which predicted the maximum at La Tola more than 20 days before the observed maximum. When late blight development was simulated on cultivar Bolona, BLIGHTSIM again showed a better fit than LB2004 to both calibration and validation data in Cutuglahua and La Tola, respectively, in 1997. To obtain good fits of BLIGHTSIM predictions to the observed data, the estimated initial inoculum was higher in Cutuglahua than in La Tola. This difference can be explained by the fact that the disease was observed before inoculation in Cutuglahua but not in La Tola [47].

Despite the excellent fit of BLIGHTSIM predictions to observed data, there are limitations to this simple simulation model. We implicitly assume that the initial inoculum consists of sporangia or zoospores, not oospores. In some cases, such as in southern Sweden, the presence of oospores in soil could initiate epidemic development earlier in the season [52]. Moreover, sporulation during the season is not explicitly modeled as it is in LATEBLIGHT and LB2004 [27,47]. Thus, spore dispersal and dilution are estimated only indirectly, while aerial spore concentrations were shown to contribute towards improved late blight predictions [13]. Future expansion of BLIGHTSIM with a spatial component may require detailed simulation of spore dispersal [19]. However, this may render the model too complex for risk assessment of late blight at a global scale. 

BLIGHTSIM was developed to predict global late blight epidemics under future climate change scenarios. Potential effects of climate change on potato late blight have been studied using various forecasting models where risk units are accumulated depending on temperatures during periods of high RH suitable for infection [6,9,18,23]. Unlike mechanistic models such as BLIGHTSIM, these models do not take into account that different stages in pathogen development respond differentially and instantaneously to changing environmental conditions. Such models can only give very broad predictions of changes in late blight severity in response to large global trends in climate change [23]. However, in the most recent Intergovernmental Panel on Climate Change (IPCC) report (namely AR5) climate change predictions have improved considerably, including the prediction of regional and inter-annual variation in average daily weather, as well as the daily temperature range [33]. 

Both average temperatures and diurnal amplitudes are expected to increase in the Andean highlands [37]. These changes in climate may have contributed to the recent increase in late blight at high altitudes [37]. Greater temperature amplitudes at relatively low average temperatures in the highlands could result in a shorter latent period compared to smaller amplitudes, and possibly in higher infection efficiency, but not in greater lesion growth and sporulation intensity [40]. Thus, the overall effect of greater amplitudes on these epidemic components cannot be predicted a priori. Another complication is that maximum temperatures are expected to occur later in the afternoon [53], so that dew periods and high RH may last longer in the morning [39]. This may enable spore release and infection to occur in the same morning, reducing the risk of spore desiccation and death due to UV radiation in the afternoon [39,41,42,50,51]. However, it is difficult to predict the effects of climate change on epidemic development based on the potential effects on individual processes in the infection cycle. A simulation model such as BLIGHTSIM could potentially better integrate the effects of climate change on various stages in the infection cycle and predict late blight development more accurately under a range of climate scenarios. Contrary to intuitive predictions, enhanced temperature amplitudes may result in reduced late blight development at low average temperatures, but not at intermediate average temperatures, as shown in this paper (Figure 5A). Thus, an increase in average temperature and temperature amplitude could indeed result in increased late blight development as observed in recent years.

Simulation models of plant disease development coupled to plant growth models can also be used for predicting the impact of climate change on the crop growth and yield as affected by plant diseases [30,44,49]. The simulation models based on LATEBLIGHT are too complex and time-consuming to be readily coupled to crop growth models. The simple SEIR model BLIGHTSIM, with a run-time of a few seconds per growing season (2000 h) and the ability to calculate AUDPC in an instant on a typical desktop computer, can be easily integrated into a potato growth model. Crop growth models generally have daily time steps, and potentially, disease progress from BLIGHTSIM could be updated daily and entered into a potato growth model to provide feedback to various physiological crop growth processes. Thus, BLIGHTSIM can play an important role in predicting the effects of climate change on potato production. A combined BLIGHTSIM–potato model will also allow comparison of adaptation strategies and their economic impacts, so that farming communities can better adjust to the impending changes in their environment. 

## 4. Materials and Methods 

### 4.1. Model Assumptions

The model BLIGHTSIM is assumed to consist of four basic compartments: healthy (H), latently infected (Lx), infectious (I) and removed (R). An infection site is the basic unit of the model and is assumed to be 1mm^2^ of leaf surface area. The total set of sites is assumed to be constant throughout the epidemic (which usually takes 3–4 weeks). Susceptible sites are randomly distributed over an average plot area of 22 m^2^ planted to potatoes, which was the plot size of experiments carried out by [47] in Ecuador. The leaf area index (LAI) has a constant value of 2 (m^2^.m^−2^) [54] and we assume that vertical position in the canopy does not affect epidemic processes. A single leaf has a maximum of 2000 infection sites. Stems are not considered to have susceptible sites. 

The minimum latent period is 57 h for the US-23 isolate used in a growth chamber study [40]; in the model, the value ranges from 57.5 to 118.5 h (with an average of 77 h) depending on temperature. New infections are directly related to the proportion of infectious sites. No distinction is made between infection by zoospores or sporangia. The relative reproduction rate (RRR) combines the effects of hourly spore production (HSP) and the dilution factor (DILFAC). HSP is 45 at 15 ± 5 °C and lower at more extreme temperatures [40]. The dilution factor is assumed to be 0.01, meaning that 99 percent of the spores do not land on susceptible sites. Thus, the initial RRR for field calibration purposes was 0.045. The estimated RRR value was multiplied by two reduction factors to account for the infection efficiency as related to temperature and RH (see below).

New latent sites originate through two mechanisms: infection by spores produced at infectious sites, and lesion growth from latent sites during the last day before they become infectious. Lesion growth is not modeled spatially, because the model does not keep track of individual leaves and their locations. Therefore, “lesion growth” will be placed between single quotation marks in this paper. The transition from healthy into latent sites can result from two processes: infection by spores and “lesion growth”. Although infection and “lesion growth” are not modeled spatially, they can be envisioned as follows. New latent sites infected by spores are divided into two categories: initial latent sites (during most of the latent period) and last-day latent sites, which contribute to “lesion growth”. Newly infected rings around the lesions move into the infectious category in one day under optimal temperature conditions, in a similar way as in the simulation model by [49]. Each lesion grows with a constant radial lesion growth rate from the latent edge of each lesion. This implies that the area of each lesion grows approximately at a quadratic declining rate (the rates were optimized based on calibration with growth chamber data; see supporting material, Table S2). To avoid “lesion growth” over the leaf margin, “lesion growth” stops after three days at optimal temperatures (transfer of sites from H into the state variables Latent5 and Latent6 is assumed to be 0 at temperatures of 17 °C or above). After a site moves through latency, it becomes infectious. The infectious period of a sporulating ring of a lesion (internal to the ring of latent tissue) is assumed to be 24 h [27].

### 4.2. Basic Model Structure

BLIGHTSIM is a modified SEIR compartmental model (Figure 1). The compartments are healthy sites (H), latent sites (Lx), infectious sites (I) and removed sites (R). Latently infected sites are divided into 7 latent compartments: L0 is formed from infection of H by spores and turns into L1 towards the end of the latent period; the other latent compartments (L2, L3, L4, L5 and L6) originate from “lesion growth” on five successive days. 

The model starts with the proportion of healthy susceptible sites and latently infected sites at 7:00 am in the morning. Healthy susceptible sites decrease in two ways: (i) through new infection determined by the number of infectious sites, and (ii) through the “growth” of the latently infected sites. The rate of transition from latent to infectious state is determined by latency progression rate (LPR), which is the inverse of the latent period under optimal conditions. Two latency progression rates are distinguished (Table 3): LPR0 is 1/53 h-1, and LPR is 1/24 h-1, based on the idea that latent lesions contribute to “lesion growth” during the last 24 h before they become infectious. Thus, the total latent period is 77 h under optimal conditions. Under suboptimal temperature conditions, latency development rates are reduced by a temperature function. For calibration of the model with data from a growth chamber [40], where leaflets were inoculated on day 0, a delay function was needed by setting LPR0 to 0 for the duration of the latent period (minus one day). This delay function was not needed for simulation under field conditions where generations would overlap. For the transition from the last stage of latency to the infectious state, LPR was multiplied by a temperature factor as well as a RH factor. The rate of change from the infectious to the removed state is determined by the removal rate, 1/24 h-1, which is the inverse of the infectious period. 

BLIGHTSIM uses hourly temperature and RH data as input to simulate the relative disease severity (as proportion of all sites), which is calculated as the sum of the proportions of infectious and removed sites. Temperature and RH are the driving variables of the system which affect the latency progression and relative “lesion growth” rates, as well as RRR. At the end of the simulation run, the area under the disease progress curve (AUDPC) is calculated by adding the hourly relative severity values [55]. The program was implemented using the R programming environment (version 3.2.2, Auckland University, Auckland, New Zealand) with hourly time steps and using Runge–Kutta methods for integration.

### 4.3. Effects of Environmental Conditions

#### 4.3.1. Effect of Temperature on Relative SporulationXInfection and Derivation of Function f1

A four-parameter thermodynamic model [56] was used to describe the combined effect of temperature on relative sporulation and infection (Equation (1)). Independent sporulation and infection efficiency curves described in [40] for an isolate of *P*. *infestans* clone US-23 were merged by multiplying relative sporulation and infection efficiency at each temperature to produce a single response curve (Figure 2A). The four-parameter model was as follows:

(1) $$r\;\left(T\right)\;=\frac{\rho 25\;\frac{T+273.2\;}{298}\;exp\left[\frac{\Delta HA}{1.987}\left(\frac{1}{298}-\frac{1}{T+273.2}\right)\right]}{1+exp\left[\frac{\Delta HH}{1.987}\;\left(\frac{1}{T1/2H}-\frac{1}{T+273.2}\right)\right]}$$

where ρ25 = development rate at 25 °C assuming no enzyme activation;

T = temperature;

ΔHA = Enthalpy of activation of the reaction catalyzed by the enzyme;

ΔHH = Change in enthalpy associated with high temperature inactivation of the enzyme;

T1/2H = Temperature at which the enzyme is half active and high temperature inactive.

All parameters and their values are described in Table S1. The parameter values were obtained by fitting the data to the model in SAS version 9.4 (SAS Institute Inc., Cary, NC, USA).

#### 4.3.2. Effect of Relative Humidity on Sporulation and Derivation of Function f2

Function f2 (Equation (2)) (Figure 2B) was derived from the sporulation data measured 10 days after inoculation on detached potato leaflets at 0.3 mm.s^−1^ air speed, at 15 °C and 80%, 85%, 90%, 95% or 100% RH [48]. To derive the parameters for function f2, the sporulation data were logit-transformed and fitted to a linear regression model is SAS version 9.4.

f2 = 1/(1 + (1.86e+16)*exp(−0.43482*RH))(2)

#### 4.3.3. Effect of Temperature on Relative “Lesion Growth” and Derivation of Function f3

The relative lesion growth rates for 3 to 5 consecutive days (depending on temperature) were derived using the data from growth chamber experiments for the US-23 isolate as described under “Estimation of relative lesion growth rate” (Section 4.5). In the model, these rates were adjusted for the effect of temperature on “lesion growth” using function f3. To obtain function f3, the increase in lesion radius per hour was calculated at different constant temperatures and fitted to the thermodynamic model (Equation (1), Figure 2C). The estimated parameter values (Table S1) were used in the simulation model. 

#### 4.3.4. Effect of Temperature on Latency Progression Rate and Derivation of Function f4

The inverse of the latent periods for cultivar Red LaSoda and an isolate of US-23 at a range of constant temperatures [40] were fitted to the same thermodynamic model. This resulted in a response curve for the relative latency progression rate versus temperature, function f4 (Equation (1), Figure 2D). Parameter estimates for function f4 are given in Table S1.

### 4.4. Model Equations

The change in each state variable of the system is governed by the following equations (the code is given in Table S3).

dH = ((−RRR )*f1*f2*(H*I)) − (RLGR1*f3*H*L1) − (RLGR2*f3*H*L2) − (RLGR3*f3*H*L3) − (RLGR4*f3*H*L4) − (RLGR5*f3*H*L5))(3)

dL0 = ((RRR)*f1*f2*(H*I)) − (L1*LPR0*f4))(4)

dL1 = ((L0*LPR0*f4)) − (L1*LPR*f4))(5)

dL2 = (RLGR1*f3*H*L1) − (L2*LPR*f4))(6)

dL3 = (RLGR2*f3*H*L2) − (L3*LPR*f4))(7)

dL4 = (RLGR3*f3*H*L3) − (L4*LPR*f4))(8)

dL5 = (RLGR4*f3*H*L4) − (L5*LPR*f4))(9)

dL6 = (RLGR5*f3*H*L5) − (L6*LPR*f4))(10)

dI = (L1*LPR*f4) + (L2*LPR*f4) + (L3*LPR*f4) + (L4*LPR*f4) + (L5*LPR*f4) + (L6*LPR*f4) − (REMRATE*I))(11)

dR = ((REMRATE)*I))(12)

dY = dI + dR(13)

where dH is changes in healthy sites, dL0 to dL6 are changes in latent sites and dI, dR and dY are changes in infectious, removed and sum of infectious and removed sites, respectively.

f1 = a reducing function that describes the effect of temperature on sporulation and infection (Table S1);
f2 = a reducing function that describes the effect of RH on sporulation;
f3 = a reducing function that describes the effect of temperature in radial “lesion growth”;
f4 = a reducing function that describes the effect of temperature on the latency progression rate.

For detailed and completed definition of parameters refer to Table 3.

### 4.5. Estimation of Relative Lesion Growth Rate

Under optimal temperature conditions, a lesion will reach the margin of a leaf in 72 h (3 days). Under suboptimal conditions, this process can take 120 h (5 days). To avoid growth beyond the leaf margin, the number of parallel latent states (rings of “lesion growth”) is limited to 3, 4 or 5, depending on temperature conditions. The radius of each lesion grows linearly, so that the relative lesion growth rate (in terms of increase in latent sites relative to the previously formed latent sites) declines according to a power function (Table S1). However, the relative lesion growth rates could not be described by a simple equation, as they were optimized to fit the growth chamber data. The relative lesion growth rates were calculated in Excel (Microsoft Office Professional Plus 2013, Microsoft Corp., Redmond, WA, USA) as described in the supporting information (Tables S1 and S2). First, the hourly increase in radius and lesion area was plotted using the data for the US-23 isolate [40]. Then, the derivative of the increase in lesion area over time was calculated. Further, this derivative was divided by the lesion area to calculate the relative lesion growth rate. Because five parallel latency pathways were included in the model, one for each day of “lesion growth”, the decline curve for relative lesion growth rate was divided into five discrete sections (for a maximal 5-day period of “lesion growth” per leaf), and the average daily relative growth rates were calculated for five consecutive 24-h periods (Tables S1 and S2). To account for declining relative lesion growth rates within each day, average daily lesion growth rates were multiplied by a weighting factor. The highest weight was given to the first four hours of each day (0.9) and less weight (0.02) was assigned to each subsequent four-hour interval (so that the sum of the weights was 1.0 each day). 

No distinction could be made between lesion growth rates during the day or night, since nothing has been reported about relative lesion growth rates in the light versus the dark. 

### 4.6. Driving Variables

The model was designed for easy application to new environmental data sets and uses hourly temperature and RH data in an Excel spread sheet (“csv” format) as driving variables. For model calibration the hourly values were the same as those attained in the growth chamber experiments [40]. Oscillating temperatures in the growth chamber were achieved by using a modified sine-wave equation to set the temperature controls of the growth chamber. The equation was [57]:

TH = A* Sin (Radians (15*H + 210)) + MT (14)

where TH = temperature at hour H, A = amplitude (Tmax−Tmin)/2, H = time in 24 h, M = mean temperature, Tmax = maximum temperature in 24 h, Tmin = minimum temperature in 24 h. The RH of the chamber was set for 60% to 70% during the day and 90% to 95% at night. Lights were turned on during the day and turned off at night. Each day started at 7:00 am and ended at 7:00 pm [40].

For scenario testing, the temperature data entered into the model were obtained from the same sinewave as used for the growth chamber experiment. A modified version of this sine-wave equation was used to generate RH data:

RHH = A* Sin (Radians (15*H + 30)) + Mrh(15)

where RHH = relative humidity at hour H, A = amplitude (RHmax−RHmin)/2, H = time in 24 h, Mrh = mean relative humidity, RHmax = maximum RH in 24 h, RHmin = minimum RH in 24 h.

### 4.7. Model Calibration with Growth Chamber Data

The model was calibrated with disease progress curves for “lesion growth” of an isolate of *P*. *infestans* clone US-23 in a growth chamber set at 21 different average temperature and amplitude combinations [40]. Six potato leaflets (about 2000 mm^2^ each) had been inoculated with *P*. *infestans* zoospores for each average temperature and amplitude combination, and infection efficiency, incubation and latency progression, “lesion growth” and sporulation were followed over time. Re-infection from sporangia or zoospores was avoided by terminating each experiment 168 h after inoculation. Disease progress curves were constructed taking the incubation period and observed “lesion growth” into account. The model was run for 168 h starting at 7:00 am on the day of inoculation. Because secondary cycles were avoided, the number of initial infectious sites and RRR were set to zero during model calibration with the growth chamber data. 

The model was initiated with particular proportions of latent sites in the L0 compartment for each temperature (Table 1). The initial proportion of L0 sites within the inoculated area per leaf was determined by dividing the estimated initial lesion area by the contact area of the inoculation droplet (14 mm^2^). The proportion of initial latent sites (L0) per total leaf area was estimated for each average temperature and amplitude combination taking the ratio of inoculated to total leaf area into account. The proportion of healthy sites at the starting hour was calculated by subtracting the proportion of latent sites from 1.0. The proportions of initial L0 sites were optimized to obtain a good fit of the simulated to the observed disease progress curves at each average temperature and amplitude combination. The rationale for this was that the infection efficiency was affected by temperature [40].

The simulated relative diseased area was considered to be the sum of the proportions of infectious (I) and removed (R) sites. Simulated and observed relative disease severities were plotted over time for each of the 21 average temperature and amplitude combinations. Simulated and observed relative disease severities were also plotted against each other and linear regression equations were calculated in Microsoft Excel 2013. The slopes of the regression lines were compared to 1.0, and the R^2^ was considered as a measure of the goodness of fit (Table 1). 

### 4.8. Model Calibration and Validation with Field Data

Data preparation: to test the model under field conditions, potato late blight severity (% symptomatic leaf area per plot) and hourly temperature and RH data recorded in six field experiments were obtained for two locations in Ecuador, Cutuglahua and La Tola, in 1997 and 1998 [47]. For La Tola region, the temperature and humidity data were missing for 16 days during April and May in 1997, and 13 days during April in 1998. To estimate the temperature and RH on missing dates, the data for March 1997 and 1998 were acquired from a weather station in the vicinity of La Tola [58]. The downloaded data were regressed linearly on the La Tola data obtained from the field site in Ecuador. The correlations between the two data sets were positive (R^2^ = 0.35 and 0.58 for temperature and RH, respectively). After including the data estimated from regression, the data covered the period from inoculation or natural initial infection to the end of the epidemics in each year: 1465 h and 2160 h for Cutuglahua and 1392 h and 1735 h for La Tola in 1997 and 1998, respectively.

Simulations: two late blight susceptible cultivars Gabriela and Bolona were selected to calibrate and validate the BLIGHTSIM model (Table 2). The inoculation dates mentioned in [47] were used as the starting dates of the simulation runs for the field. The starting times were 7.00 am, similar to the inoculation time in the growth chamber experiment and the simulation starting times used for fitting the growth chamber data. The model was calibrated for each cultivar and location in 1997 to find the best initial inoculum proportion (L0) and RRR. The values of the other parameters such as RLGR, LPR were the same as those used for simulation of the growth chamber data. The weather data of the next year (1998) were used to validate the model simulations based on the estimated initial inoculum and RRR obtained in the calibration process. Goodness of fit (R^2^) was evaluated by regressing the observed field data on the simulated data. Unlike the model fitting with growth chamber data when a delay was used until the end of the latent period, no delay time was used for calibration and testing the model with the field data. Instead, the latent and infectious periods were accounted for by taking the inverse of these periods as latency and infectiousness development rates [45].

### 4.9. Scenario Testing

The BLIGHTSIM model was used to investigate two sets of scenarios. AUDPC values were calculated by integrating hourly severity values within BLIGHTSIM using the R code of [55], and these were used as the response variable for all scenarios. The purpose of the first scenario set was to investigate if there was an interaction between average temperature and temperature amplitude with respect to the effects on AUDPC and to check if the temperature amplitude was more important than the average temperature or vice versa. First, hourly temperature and RH data were created (1200 h) using the same sine-wave equations as described earlier (see Section 4.4). 

Simulations were evaluated for 1200 h time courses for all combinations of seven average temperatures (10 °C, 12 °C, 15 °C, 17 °C, 20 °C, 23 °C and 27 °C) and three temperature amplitudes (0 °C, 5 °C and 10 °C). The oscillations in RH were so that the maximum RH was 100% at 4:00 am and the minimum 50% at 4:00 pm. Three sets of parameter values of RRR, L0, RLGRx, LPRx and INFR (Infection Rate) that were estimated during calibration of the Cutuglahua data in 1997 (cultivar Gabriella), the La Tola data in 1997 (cultivar Gabriella), and the Cutuglahua data in 1997 (cultivar Bolona) were used in simulations, resulting in three independent replications for each of the average temperatures and temperature amplitudes. For this scenario, the model was run 63 times (seven average temperatures, three amplitudes and three locations) and the AUDPC values for each epidemic were calculated and compared. 

The aim of the second scenario set was to investigate how the epidemics are affected when the model is initialized with latent or infectious sites and if the starting hour of the simulation has a substantial effect on disease progress. Temperature oscillations had an average temperature of 17 °C with an amplitude of 5 °C. The RH oscillations were as described in the previous paragraph. The same three sets of parameter estimates (RRR, L0, RLGRx, LPRx and INFR) were used as mentioned in the previous paragraph. The model runs were initiated with LAT0 or INF, starting at 7:00 am, 1:00 pm, 7:00 pm or 1:00 am for 1200 h each run. In total, the model was run 24 times (two types of initial sites, four starting times and three model runs).

While statistical analysis of simulation results can be misinterpreted [59], we include statistical analyses here because the objective was to investigate potential interactions between two factors in each scenario. The variation in the three simulation replicates was generated through use of three sets of parameters derived from three independent field studies, unlike the simulations discussed by [59]. Because the AUDPC data were expected to be auto-correlated, they were analyzed by repeated measures regression analysis (PROC GLM in SAS 9.4) to test for an interaction between temperature average and amplitude for scenario one, and between starting time and initialization sites (LAT0 or INF) for scenario two. The AUDPC values for different average temperatures or starting times were subjected to polynomial regression as part of the repeated measures analysis.

## Figures and Tables

**Table 1:** Initial values of the proportions of latently infected sites (L0), coefficients of determination (R^2^) and slope at different average temperature and amplitude combinations for the BLIGHTSIM model.

Temperature ^a^	±0 °C ^b^			±5 °C			±10 °C		
	L0^c^	R^2^	Slope	L0	R^2^	Slope	L0	R^2^	Slope
10	0.0078	0.934	1.02	0.025	0.970	1.01	0.0088	0.997	1.00
12	0.0312	0.991	1.02	0.056	0.996	1.02	0.0122	0.995	0.999
15	0.034	0.994	1.03	0.0280	0.997	1.02	0.0101	0.997	1.01
17	0.0215	0.996	1.03	0.019	0.996	1.06	0.0158	0.997	1.02
20	0.0122	0.995	1.04	0.0055	0.996	1.02	0.030	0.995	1.03
23	0.0280	0.966	1.10	0.006	0.997	1.04	0.037	0.988	1.04
27	0.0195	0.994	1.04	0.0036	0.998	1.02	0.016	0.989	1.03

^a^ Average temperature in °C. ^b^ Amplitudes of daily temperature oscillations. ^c^ The initial values of all other state variables (except for healthy sites) are zero.

**Table 2:** Estimates of the relative reproductive rate (RRR) and the proportion of initial inoculum (L0) obtained by calibration of the BLIGHTSIM model for two different locations, years and cultivars in Ecuador.

Location	Year	Cultivar	RRR	L0	R^2^	
Cutuglahua	1997	Bolona	0.052	0.0007	0.977	
Cutuglahua	1997	Gabriela	0.049	0.0007	0.976	
Cutuglahua	1998	Gabriela	0.049	0.0001	0.991	
La Tola	1997	Bolona	0.052	0.000001	0.961	
La Tola	1997	Gabriela	0.049	0.000001	0.875	
La Tola	1998	Gabriela	0.049	0.000001	0.897	

**Table 3:** Estimates of the relative reproductive rate (RRR) and the proportion of initial inoculum (L0) obtained by calibration of the BLIGHTSIM model for two different locations, years and cultivars in Ecuador.

State Variables	Description (Units)	Initial Values
H	Healthy susceptible sites	Table 1
L_x_	Latently infected sites	
I	Infectious sites	0
R	Removed sites	0
Y	Sum of infectious and removed sites	0
Driving variables		
T	Hourly temperature (°C)	0-37
RH	Hourly relative humidity (%)	60-95
Parameters		
LPR0	Latency progression rate 1 (h^−1^)	1/53 at 23 °C
LPR1	Latency progression rate 2 (h^−1^)	1/24 at 23 °C
RLGR1	Relative lesion growth rate 1(h^−1^)	0.9544
RLGR2	Relative lesion growth rate 2(h^−1^)	0. 07336
RLGR3	Relative lesion growth rate 3(h^−1^)	0. 03888
RLGR4	Relative lesion growth rate 4(h^−1^)	0.02648
RLGR5	Relative lesion growth rate 5(h^−1^)	0.02009
REMRATE	Relative rate of removal (h^−1^)	1/24
HSP	Relative hourly spore production (h^−1^ )	45
DILFAC	Dilution factor (-)	0.01
RRR ( = HSP*DILFAC)	Relative reproduction rate (h^−1^)	0

## Supplementary Materials

The following are available online at https://www.mdpi.com/2076-0817/9/8/659/s1, Figure S1: Observed (dots) and simulated (continuous line) disease progress curves of an isolate of *Phytophthora infestans* clone US-23 at different constant temperatures, Figure S2: Observed (dots) and simulated (continuous line) disease progress curves of an isolate of *Phytophthora infestans* clone US-23 at different oscillating temperatures, Figure S3: Observed (dots) and simulated (continuous line) disease progress curves of an isolate of *Phytophthora infestans* clone US-23 at different oscillating temperatures, Figure S4: The effect of initiation of the simulation with proportion of latent or infectious sites and of the starting time of day on the average simulated AUDPC values (three replications) of late blight development over 1200 h, Table S1: Estimated parameter values for the temperature functions (f1, f3 and f4) obtained by fitting a thermodynamic model to epidemic components of an isolate of *Phytophthora infestans* clone US-23 on potato cv. Red LaSoda and R code on Estimation of relative lesion growth rate; Table S2: Calculations of the relative lesion growth rate for the simulation model BLIGHTSIM, with means calculated every 24 h; Table S3: The annotated BLIGHTSIM script in R.
