# Supplementary material for: BLIGHTSIM: A New Potato Late Blight Model Simulating the Response of Phytophthora infestans to Diurnal Temperature and Humidity Fluctuations in Relation to Climate Change
Source: Pathogens. 2020 Aug 15;9(8):659. doi: 10.3390/pathogens9080659 (PMC7459445; doi:10.3390/pathogens9080659)
Supplement: Supplementary file 1 [file pathogens-09-00659-s001.pdf]

Supporting information

BLIGHTSIM: A New Potato Late Blight Model Simulating the Response of *Phytophthora infestans* to Diurnal Temperature and Humidity Fluctuations in Relation to Climate Change

Hossein A. Narouei-Khandan [1,2,3,*], Shankar K. Shakya [1], Karen A. Garrett [1,2,4], Erica M. Goss [1,2], Nicholas S. Dufault [1], Jorge L. Andrade-Piedra [5], Senthold Asseng [6], Daniel Wallach [7] and Ariena H.C van Bruggen [1,2]

[1] Department of Plant Pathology, University of Florida, 1450 Fifield Hall, P.O. Box 110680, Gainesville, FL 32611-0680, USA; ahcvanbruggen@ufl.edu (A.H.C.v.B.); hossein.khandan@mpi.govt.nz (H.A.N.-K.); sshakya@ballhort.com (S.K.S.); emgoss@ufl.edu (E.M.G.); nsdufault@ufl.edu (N.S.D.)
[2] Emerging Pathogens Institute, University of Florida, Gainesville, 2055 Mowry Road, P.O. Box 100009, Gainesville, FL 32610, USA; karengarrett@ufl.edu (K.A.G.)
[3] Ministry for Primary Industries, PO Box 2526, Wellington 6146, New Zealand
[4] Food Systems Institute, University of Florida, P.O. Box 110180, Gainesville, FL 32611-0180, USA
[5] International Potato Center (CIP) and CGIAR Research Program on Roots Tubers and Bananas (RTB), P.O. Box 1558, Lima 12, Peru; j.andrade@cgiar.org (J.L.A.-P.)
[6] Department of Agricultural and Biological Engineering, University of Florida, 224 Frazier Rogers Hall, P.O. Box 110570, Gainesville, FL 32611-0570, USA; sasseng@ufl.edu (S.A.)
[7] Institut National de la Recherche Agronomique (INRA), UMR AGIR, BP 52627, 31326 Castanet Tolosan Cedex, France; Daniel.wallach@inrae.fr(D.W.)
* Correspondence: hossein.khandan@mpi.govt.nz

Table S1. Estimated parameter values for the temperature functions (f1, f3 and f4) obtained by fitting a thermodynamic model to epidemic components of an isolate of *Phytophthora infestans* clone US-23 on potato cv. Red Lasoda [40]. A logistic regression model was fitted to calculate the f2 function[1].

Parameter	Parameter description	Sporulation*inf. eff. vs. temperature (f1)	Radial lesion growth rate vs. temperature (f3)	Relative latency progression rate vs. temperature (f4)
$\rho 25$	Development rate at 25°C assuming no enzyme activation	1.013*1010	2559.1	1.7168
ΔHA	Enthalpy of activation of the reaction catalyzed by the enzyme	292131	-716288	14275.4
ΔHH	Change in enthalpy associated with high temperature inactivation of the enzyme	331573	-744720	49087.1
$T1/2H$	Temperature at which the enzyme is half active and high temperature inactive	284.9	299.8	298.85

[1] f2 = 1/(1+(1.86e+16)*exp(-0.43482*RH))

f3<-(2559.1*((T+273.2)/298)*exp((-716288/1.987)*(1/298-1/(T+273.2)))/(1+(exp((-744720/1.987)*(1/299.8-1/(T+273.2))))))

=

$\rho 25 *$

Table S2. Calculations of the relative lesion growth rate for the simulation model BLIGHTSIM, with means calculated every 24 hours.

Radius (mm)	Hr	Area pi (r2)	Derivative	Derivative /area	Mean relative rate from derivative	Weighted mean relative rate from derivative
0.0444	1	0.00619	0.01239	2		
0.0888	2	0.024773	0.024773	1		
0.1332	3	0.055739	0.037159	0.66666667		
0.1776	4	0.099092	0.049546	0.5		
0.222	5	0.154831	0.061932	0.4		
0.2664	6	0.222956	0.074319	0.33333333		
0.3108	7	0.303468	0.086705	0.28571429		
0.3552	8	0.396366	0.099092	0.25		
0.3996	9	0.501651	0.111478	0.22222222		
0.444	10	0.619322	0.123864	0.2		
0.4884	11	0.74938	0.136251	0.18181818		
0.5328	12	0.891824	0.148637	0.16666667		
0.5772	13	1.046655	0.161024	0.15384615		
0.6216	14	1.213872	0.17341	0.14285714		
0.666	15	1.393476	0.185797	0.13333333		
0.7104	16	1.585465	0.198183	0.125		
0.7548	17	1.789842	0.21057	0.11764706		
0.7992	18	2.006605	0.222956	0.11111111		
0.8436	19	2.235754	0.235343	0.10526316		
0.888	20	2.47729	0.247729	0.1		
0.9324	21	2.731212	0.260115	0.0952381		
0.9768	22	2.997521	0.272502	0.09090909		
1.0212	23	3.276216	0.284888	0.08695652		
1.0656	24	3.567297	0.297275	0.08333333	0.314663181	0.954426248
1.11	25	3.870765	0.309661	0.08		
1.1544	26	4.18662	0.322048	0.07692308		
1.1988	27	4.514861	0.334434	0.07407407		
1.2432	28	4.855488	0.346821	0.07142857		
1.2876	29	5.208502	0.359207	0.06896552		
1.332	30	5.573902	0.371593	0.06666667		
1.3764	31	5.951689	0.38398	0.06451613		
1.4208	32	6.341862	0.396366	0.0625		
1.4652	33	6.744422	0.408753	0.06060606		
1.5096	34	7.159368	0.421139	0.05882353		
1.554	35	7.5867	0.433526	0.05714286		
1.5984	36	8.026419	0.445912	0.05555556		
1.6428	37	8.478524	0.458299	0.05405405		
1.6872	38	8.943016	0.470685	0.05263158		

1.7316	39	9.419895	0.483072	0.05128205		
1.776	40	9.909159	0.495458	0.05		
1.8204	41	10.41081	0.507844	0.04878049		
1.8648	42	10.92485	0.520231	0.04761905		
1.9092	43	11.45127	0.532617	0.04651163		
1.9536	44	11.99008	0.545004	0.04545455		
1.998	45	12.54128	0.55739	0.04444444		
2.0424	46	13.10486	0.569777	0.04347826		
2.0868	47	13.68083	0.582163	0.04255319		
2.1312	48	14.26919	0.59455	0.04166667	0.05690325	0.073362049
2.1756	49	14.86993	0.606936	0.04081633		
2.22	50	15.48306	0.619322	0.04		
2.2644	51	16.10858	0.631709	0.03921569		
2.3088	52	16.74648	0.644095	0.03846154		
2.3532	53	17.39677	0.656482	0.03773585		
2.3976	54	18.05944	0.668868	0.03703704		
2.442	55	18.7345	0.681255	0.03636364		
2.4864	56	19.42195	0.693641	0.03571429		
2.5308	57	20.12179	0.706028	0.03508772		
2.5752	58	20.83401	0.718414	0.03448276		
2.6196	59	21.55861	0.7308	0.03389831		
2.664	60	22.29561	0.743187	0.03333333		
2.7084	61	23.04499	0.755573	0.03278689		
2.7528	62	23.80676	0.76796	0.03225806		
2.7972	63	24.58091	0.780346	0.03174603		
2.8416	64	25.36745	0.792733	0.03125		
2.886	65	26.16637	0.805119	0.03076923		
2.9304	66	26.97769	0.817506	0.03030303		
2.9748	67	27.80139	0.829892	0.02985075		
3.0192	68	28.63747	0.842279	0.02941176		
3.0636	69	29.48594	0.854665	0.02898551		
3.108	70	30.3468	0.867051	0.02857143		
3.1524	71	31.22005	0.879438	0.02816901		
3.1968	72	32.10568	0.891824	0.02777778	0.033501082	0.038888711

Etc.

Table S3. The annotated BLIGHTSIM script in R.

```
rm(list=ls())
#BLIGHTSIM model parameters include:
#RRR=Relative Reproductive Rate
#LPR1=latency progression rate1
#RLGP1=relative lesion growth rate1
#REMRATE=relative rate of removal
##############################################################################
#Adding excel file (hourly weather data) by browsing
tempdata=read.csv(file.choose(),h=T)

##############################################################################
Thour=tempdata$t
RHhour=tempdata$RH
weather=data.frame(cbind(Thour,RHhour))
head(weather)

# Define Relative Reproductive Rate, which depends on the cultivar
RRR<-0.049
#Latent Progression Rates
LPR0<-1/96
LPR<-1/24          # latent progression rate1

# Relative lesion growth rate based on weighted average of hourly differences for US23
LGR1<-0.95441
LGR2<-0.07336
LGR3<-0.03888
LGR4<-0.02648
LGR5<-0.02009

# Removal rate
REMRATE=1/24
# Starting hour
shour=1
# Total number of hours for field or growth chamber
lhour=1735

######################Main function
BLIGHTSIM=function(RRR,LPR0,LPR,LGR1,LGR2,LGR3,LGR4,LGR5,REMRATE,shour,lhour,weather)
{

H=L0=L1=L2=L3=L4=L5=L6=I=R=Y=rep(NA,lhour)

############################################################################################
# Initial inoculum proportion
L0[shour]=0.000001
```

```
H[shour]=1-L0[shour]
# Proportion of infected sites
I[shour]= 0
L1[shour]=L2[shour]=L3[shour]=L4[shour]=L5[shour]=L6[shour]=R[shour]=Y[shour]=0
for(hour in shour:(lhour-1)){
# Condition: if the temperature is above 17C then LGR4 is zero, otherwise program will use the LGR$
that already was calculated and set
LGR4<-ifelse(Thour[hour]>=17, 0, 0.02648)
# See above explanation
  LGR5<-ifelse(Thour[hour]>=17, 0, 0.02009)
#LPR0<-ifelse(hour<53,0,0.018867)
#LPR0<-ifelse(hour<96,0,0.010416)
Y[hour]<-ifelse(Y[hour]>1, 1, Y[hour])
T=weather$Thour[hour]
RH=weather$RHhour[hour]

# F functions
# Effect of temperature on relative sporulation*infection and calculation of function f1
f1<-(1.013*10^10*((T+273.2)/298)*exp((292131/1.987)*(1/298-1/(T+273.2)))/(1+(exp((331573/1.987)*(1/284.9-
1/(T+273.2))))))

#Effect of relative humidity on sporulation and calculation of function f2
f2<-1/(1+(1.86e+16)*exp(-0.43482*RH))
# Effect of temperature on relative lesion growth and calculation of function f3
f3<-(2559.1*((T+273.2)/298)*exp((-716288/1.987)*(1/298-1/(T+273.2)))/(1+(exp((-744720/1.987)*(1/299.8-
1/(T+273.2))))))

# Effect of temperature on latency progression rate and calculation of function f4
f4<-(1.7168*((T+273.2)/298)*exp((14275.4/1.987)*(1/298-1/(T+273.2)))/(1+(exp((49087.1/1.987)*(1/298.85-
1/(T+273.2))))))
dH=round(((-RRR )*f1*f2*(H[hour]*I[hour]))-(LGR1*f3*H[hour]*L1[hour])-(LGR2*f3*H[hour]*L2[hour])-
(LGR3*f3*H[hour]*L3[hour])-(LGR4*f3*H[hour]*L4[hour])-(LGR5*f3*H[hour]*L5[hour]),digits=10)
dL0=round(((RRR)*f1*f2*(H[hour]*I[hour]))-(L1[hour]*LPR0*f4),digits=10)
dL1=round(((L0[hour]*LPR0*f4))-(L1[hour]*LPR*f4),digits=10)
dL2=round((LGR1*f3*H[hour]*L1[hour])-(L2[hour]*LPR*f4),digits=10)
  dL3=round((LGR2*f3*H[hour]*L2[hour])-(L3[hour]*LPR*f4),digits=10)
dL4=round((LGR3*f3*H[hour]*L3[hour])-(L4[hour]*LPR*f4),digits=10)
dL5=round((LGR4*f3*H[hour]*L4[hour])-(L5[hour]*LPR*f4),digits=10)
dL6=round((LGR5*f3*H[hour]*L5[hour])-(L6[hour]*LPR*f4),digits=10)
dI=round((L1[hour]*LPR*f4)+(L2[hour]*LPR*f4)+(L3[hour]*LPR*f4)+(L4[hour]*LPR*f4)+(L5[hour]*LPR*f4
)+(L6[hour]*LPR*f4)-(REMRATE*I[hour]),digits=10)
dR=round(((REMRATE)*I[hour]),digits=10)
dY=dI+dR
H[hour+1]=H[hour]+dH
L0[hour+1]=L0[hour]+dL0
L1[hour+1]=L1[hour]+dL1
L2[hour+1]=L2[hour]+dL2
```

```
L3[hour+1]=L3[hour]+dL3
L4[hour+1]=L4[hour]+dL4
L5[hour+1]=L5[hour]+dL5
L6[hour+1]=L6[hour]+dL6
I[hour+1]=I[hour]+dI
R[hour+1]=R[hour]+dR
Y[hour+1]=Y[hour]+dY
}
results=data.frame(hour=shour:lhour,H=H[shour:lhour], L0=L0[shour:lhour], L1=L1[shour:lhour],
L2=L2[shour:lhour], L3=L3[shour:lhour],L4=L4[shour:lhour],L5=L5[shour:lhour],
L6=L6[shour:lhour],I=I[shour:lhour], R=R[shour:lhour], Y=Y[shour:lhour])
return(results)
}
##############################################################
results=BLIGHTSIM(RRR,LPR0,LPR,LGR1,LGR2,LGR3,LGR4,LGR5,REMRATE,shour,lhour,weather)
#############################################################   Plots
# Activate by removing hashtag in each line if you want to see the chart for each phase
#par(mfrow=c(3,4))
#plot(results$hour,results$H)
#plot(results$hour,results$L0)
#plot(results$hour,results$L1)
#plot(results$hour,results$L2)
#plot(results$hour,results$L3)
#plot(results$hour,results$L4)
#plot(results$hour,results$L5)
#plot(results$hour,results$L6)
#plot(results$hour,results$I)
#plot(results$hour,results$R)
#plot(results$hour,results$Y)
#results
############### Output###############################################
# Saving the results as BLIGHTSIM_output
BLIGHSIM_output<-results

#Set a path to save the output as an excel file
setwd("put the path here")

#Up to this part it can be used for growth chamber data or field data. The next section will extract the
disease severity values at the specific hours of interest. The field disease severity (observed) were in
weekly or 10-day intervals; the exact hour of disease severity scores were needed to plot disease
severity against output of the BLIGHSIM model.
############## Write CSV in R #####################################
#Write the results as a CSV file and desired name
write.table(BLIGHSIM_output, file = "Lat_97_bolona.csv",row.names=F, na="",col.names=T, sep=",")
######################## Write only the desired rows ( to later compare with observed data)
#For the field data (Simulated), extract the desired hours and severities
read.table<-"BLIGHSIM_output"
#BLIGHSIM_output
```

```
#########For all temps
#Activate based on the field data. In this case the observed data were on different days so the
equivalent hour was extracted from simulated output file to later overlay on observed data
######Latola 97, exports desired hour's data from thousand hours of data for later use if necessary
export <- BLIGHSIM_output[c("24","240","408","672","888","1128","1368"),c("hour", "Y")]
##########Latola_98, exports desired hour's data from thousand hours of data for later use if necessary
#export <-
BLIGHSIM_output[c("72","240","408","576","744","912","1080","1248","1416","1584","1752"),c("hour", "Y")]
######Cutuglaua 97, exports desired hour's data from thousand hours of data for later use if necessary
#export <- BLIGHSIM_output[c("168","504","840","1176","1440"),c("hour", "Y")]

##########Cutuglaua _97    Emergance date
#export <- BLIGHSIM_output[c("792","1128","1464","1800","2064"),c("hour", "Y")]
##########Cutuglaua _98
# Extracts and exports only hour and disease severity at specific hours
#export <- BLIGHSIM_output[c("24","144","336","504","648","840","1008","1176","1344","1512","1656","1848",
"2136"),c("hour", "Y")]
#Setting the path to save the extracted file
setwd("put the path here ")
write.table(export, file = "Lat_97_Bolona_selected.csv",row.names=F, na="",col.names=T, sep=",")
# Browse to the excel file which includes the observed data for the field. Notice that the hours should
be the same as extracted file
field=read.csv(file.choose(),h=T)   # reads the excel file which includes extracted DS for observed data
#Plotting the simulated data in Red and adding a line to it
plot(export$hour,export$Y, col="red")
lines(export$hour,export$Y, col="red")
#Plotting the observed data in Blue
points(field$Hour,field$Observed, col="blue")
#customizing the legend
legend(150, 0.76, legend=c("Predicted", "Observed"),col=c("red", "blue"), lty=1:1, cex=0.8)

#This Part calculates the AUDPC. If needed, it can be added at the end of the "BLIGHTSIM Growth
Chamber" script or "BLIGHTSIM Field" script.
############################## CALCULATE AUPDC ################################
# Browse to the main BLIGHTSIM output which includes all hours either for growth chamber or for the
field data
tempdata=read.csv(file.choose(),h=T)
#Define the total number of hours
tempdata[1:432,]
T=tempdata$hour
DS=tempdata$Y
cbind(T,DS)
audpc <- function(DS,T){
  n <- length(T)
  meanvec <- matrix(-1,(n-1))
  intvec <- matrix(-1,(n-1))
  for(i in 1:(n-1)){
```

```
  meanvec[i] <- mean(c(DS[i],
                       DS[i+1]))
  intvec[i] <- T[i+1] - T[i]
  }
 infprod <- meanvec * intvec
  sum(infprod)
}
audpc(DS,T) -> AUDPCfinal
AUDPCfinal
# Define the output directory
setwd("put the path here")
# Write the calculated AUDPC as a CSV file
write.table(AUDPCfinal, file = "AUPDC_Latola_98_Gabriela_0_10_Sens1200hr.csv",row.names=F, na="10-27",col.names=F, sep=",")
```

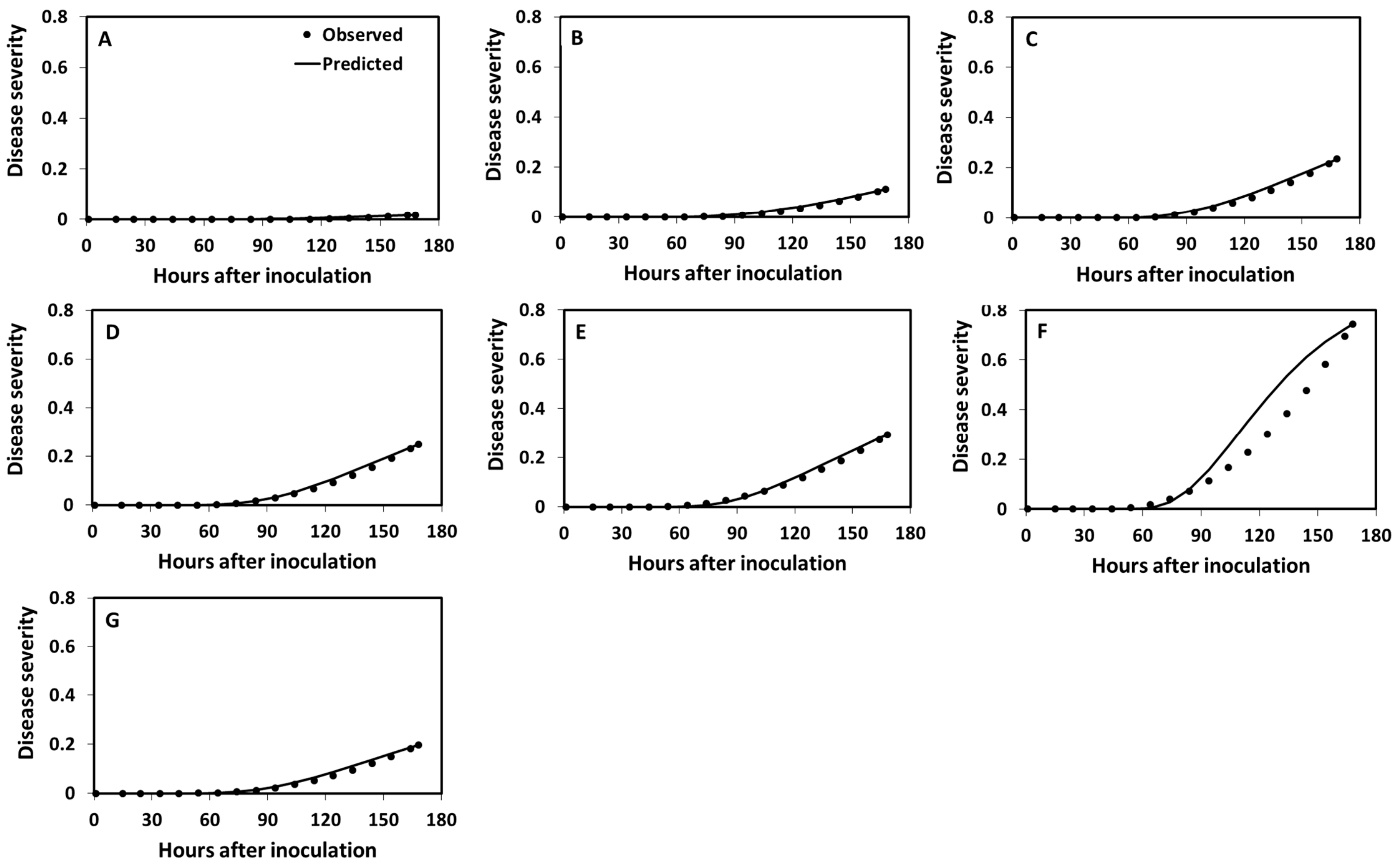

Figure S1. Observed (dots) and simulated (continuous line) disease progress curves of an isolate of *Phytophthora infestans* clone US-23 at different constant temperatures. **A**, T = 10±0°C; **B**, T = 12±0°C; **C**, T = 15±0°C; **D**, T = 17±0°C; **E**, T = 20±0°C; **F**, T = 23±0°C; and **G**, T = 27±0°C. The simulated disease progress curves were obtained with BLIGHTSIM and the observed disease severities in a growth chamber experiment at Gainesville, Florida.

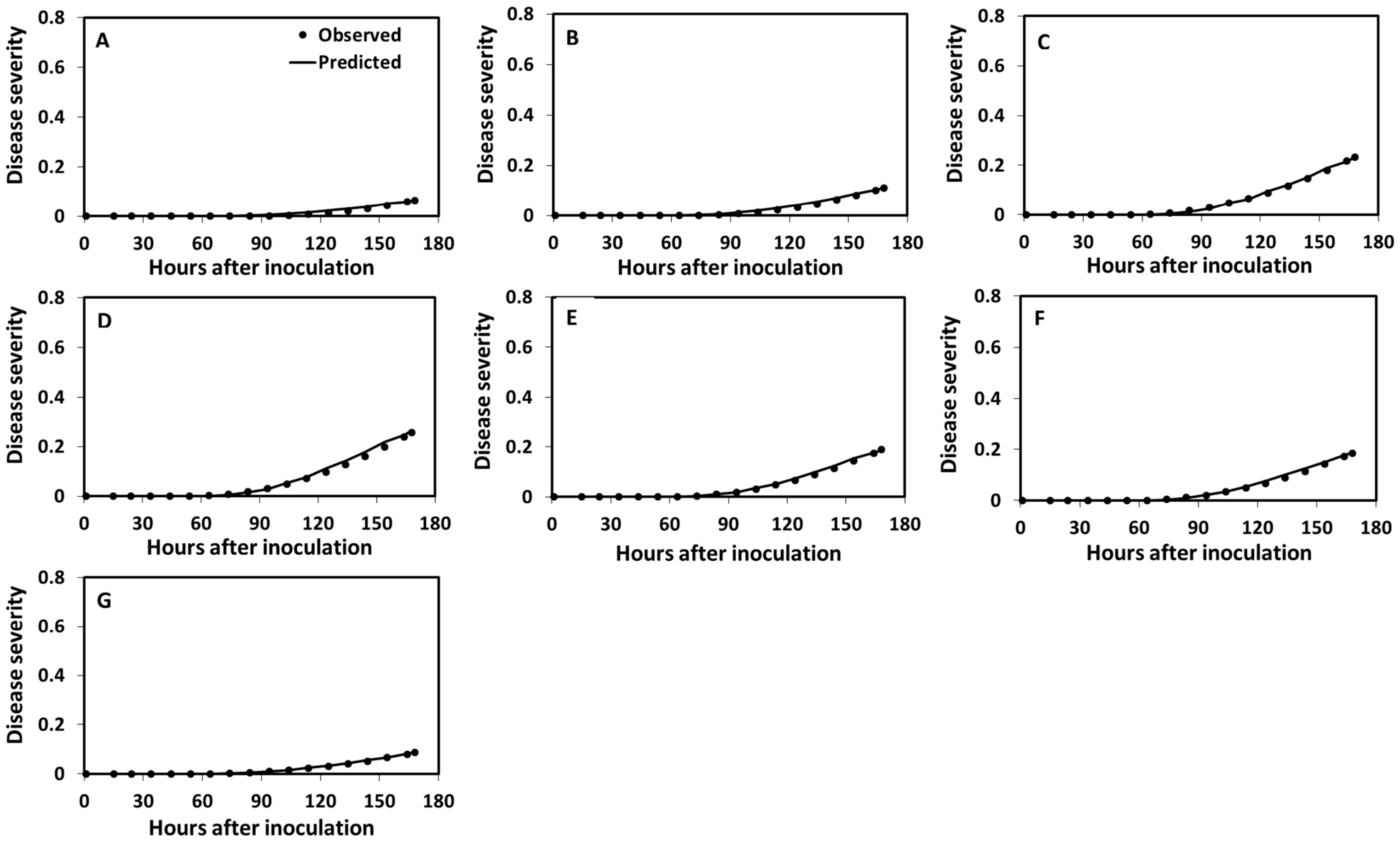

Figure S2. Observed (dots) and simulated (continuous line) disease progress curves of an isolate of *Phytophthora infestans* clone US-23 at different oscillating temperatures. **A**, T = 10±5°C; **B**, T = 12±5°C; **C**, T = 15±5°C; **D**, T = 17±5°C; **E**, T = 20±5°C; **F**, T = 23±5°C; and **G**, T = 27±5°C. The simulated disease progress curves were obtained with BLIGHTSIM and the observed disease severities in a growth chamber experiment at Gainesville, Florida.

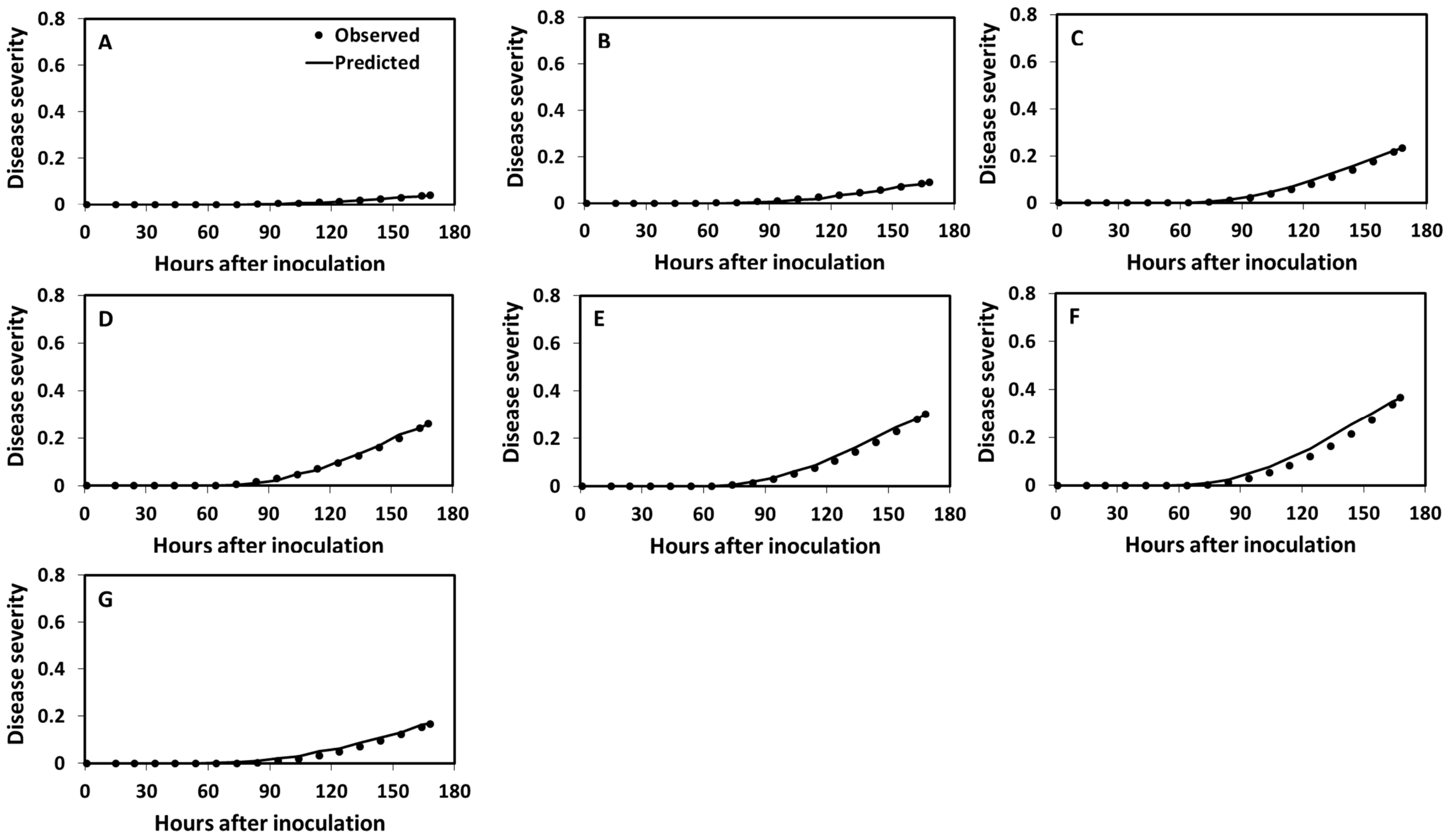

Figure S3. Observed (dots) and simulated (continuous line) disease progress curves of an isolate of *Phytophthora infestans* clone US-23 at different oscillating temperatures. A, T = 10±10°C; B, T = 12±10°C; C, T = 15±10°C; D, T = 17±10°C; E, T = 20±10°C; F, T = 23±10°C; and G, T = 27±10°C. The simulated disease progress curves were obtained with BLIGHTSIM and the observed disease severities in a growth chamber experiment at Gainesville, Florida.

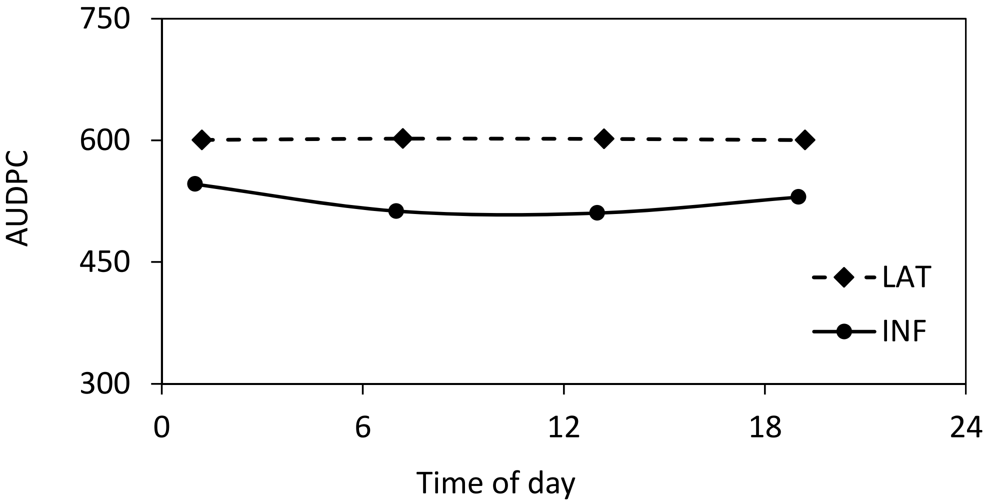

Figure S4. The effect of initiation of the simulation with proportion of latent or infectious sites and of the starting time of day on the average simulated AUDPC values (3 replications) of late blight development over 1200 h.

© 2020 by the authors. Licensee MDPI, Basel, Switzerland. This article is an open access article distributed under the terms and conditions of the Creative Commons Attribution (CC BY) license (http://creativecommons.org/licenses/by/4.0/).
